# Atg4 proteolytic activity can be inhibited by Atg1 phosphorylation

**DOI:** 10.1038/s41467-017-00302-3

**Published:** 2017-08-18

**Authors:** Jana Sánchez-Wandelmer, Franziska Kriegenburg, Sabrina Rohringer, Martina Schuschnig, Rubén Gómez-Sánchez, Bettina Zens, Susana Abreu, Ralph Hardenberg, David Hollenstein, Jieqiong Gao, Christian Ungermann, Sascha Martens, Claudine Kraft, Fulvio Reggiori

**Affiliations:** 1 Department of Cell Biology, University of Groningen, University Medical Center Groningen, A. Deusinglaan 1, 9713 AV Groningen, The Netherlands; 2 Department of Cell Biology, University Medical Center Utrecht, Heidelberglaan 100, 8564 CX Utrecht, The Netherlands; 3 Max F. Perutz Laboratories, University of Vienna, 1030 Vienna, Austria; 4 University of Osnabrück, Department of Biology/Chemistry, Biochemistry section, Barbarastrasse 13, 49076 Osnabrück, Germany

## Abstract

The biogenesis of autophagosomes depends on the conjugation of Atg8-like proteins with phosphatidylethanolamine. Atg8 processing by the cysteine protease Atg4 is required for its covalent linkage to phosphatidylethanolamine, but it is also necessary for Atg8 deconjugation from this lipid to release it from membranes. How these two cleavage steps are coordinated is unknown. Here we show that phosphorylation by Atg1 inhibits Atg4 function, an event that appears to exclusively occur at the site of autophagosome biogenesis. These results are consistent with a model where the Atg8-phosphatidylethanolamine pool essential for autophagosome formation is protected at least in part by Atg4 phosphorylation by Atg1 while newly synthesized cytoplasmic Atg8 remains susceptible to constitutive Atg4 processing.

## Abstract (web summary)

The protease Atg4 mediates Atg8 lipidation, required for autophagosome biogenesis, but also triggers Atg8 release from the membranes, however is unclear how these steps are coordinated. Here the authors show that phosphorylation by Atg1 inhibits Atg4 at autophagosome formation sites.

## Introduction

Macroautophagy (hereafter autophagy) is highly conserved among eukaryotes, and it is crucial for the maintenance of cellular homeostasis in response to cellular and environmental stresses. This pathway is also essential for a multitude of physiological processes, such as cell differentiation and defense against pathogens, and it is associated with the pathophysiology of several diseases, including cancer and neurodegeneration^1^. During autophagy, double-membrane vesicles called autophagosomes sequester cytoplasmic components and target them to lysosomes/vacuoles for degradation. The resulting metabolites are subsequently recycled back to the cytoplasm and reused for the synthesis of new macromolecules or as a source of energy^2^. In yeast, the orchestrated action of the autophagy-related (Atg) proteins at the phagophore assembly site or pre-autophagosomal structure (PAS) mediates the formation, expansion and sealing of a cistern, known as the phagophore or isolation membrane, to create an autophagosome^2^. The kinase activity of the Atg1 complex, composed by the Atg1 kinase, Atg13, Atg17, Atg29, and Atg31, is a key regulator of this process^3^.

Previous studies suggest that sealed autophagosomes cannot fuse with lysosomes/vacuoles until the Atg proteins get dissociated from autophagosomal membranes^4^, which partially depends on phosphatidylinositol-3-phosphate (PI3P) turnover^5, 6^. The Ymr1 phosphatase is pivotal in PI3P clearance on autophagosomes, which otherwise accumulate in the cytoplasm in its absence^5^. Although PI3P turnover could be sufficient to release PI3P-binding proteins, such as Atg18 and Atg21^7^, an additional mechanism is required for the dissociation of proteins such as Atg8 that are covalently conjugated to membranes. In yeast, Atg4 constitutively cleaves the C-terminal arginine of Atg8^8–10^, allowing Atg8 conjugation to the phosphatidylethanolamine (PE) in autophagosomal membranes^2^. Once conjugated to PE, Atg8 is involved in cargo selection and thought to contribute to the expansion and closure of phagophores, possibly by forming a vesicle coat^11–13^. Atg4 also deconjugates Atg8 from PE upon autophagosome completion^9, 14^. The release from its PE anchor is essential not only for Atg8 recycling but also, analogously to PI3P turnover, to allow the fusion of autophagosomes with vacuoles^9, 15, 16^. How Atg8–PE cleavage by Atg4 is timely and spatially regulated, however, is still mysterious.

In this study, we describe a novel regulatory mechanism, in which the Atg1 kinase specifically inhibits the deconjugating activity of Atg4 at the PAS, possibly contributing to the protection of the Atg8–PE pool necessary for autophagosome biogenesis.

## Results

### Atg1 phosphorylates Atg4 and inhibits autophagy

**Fig. 1:**
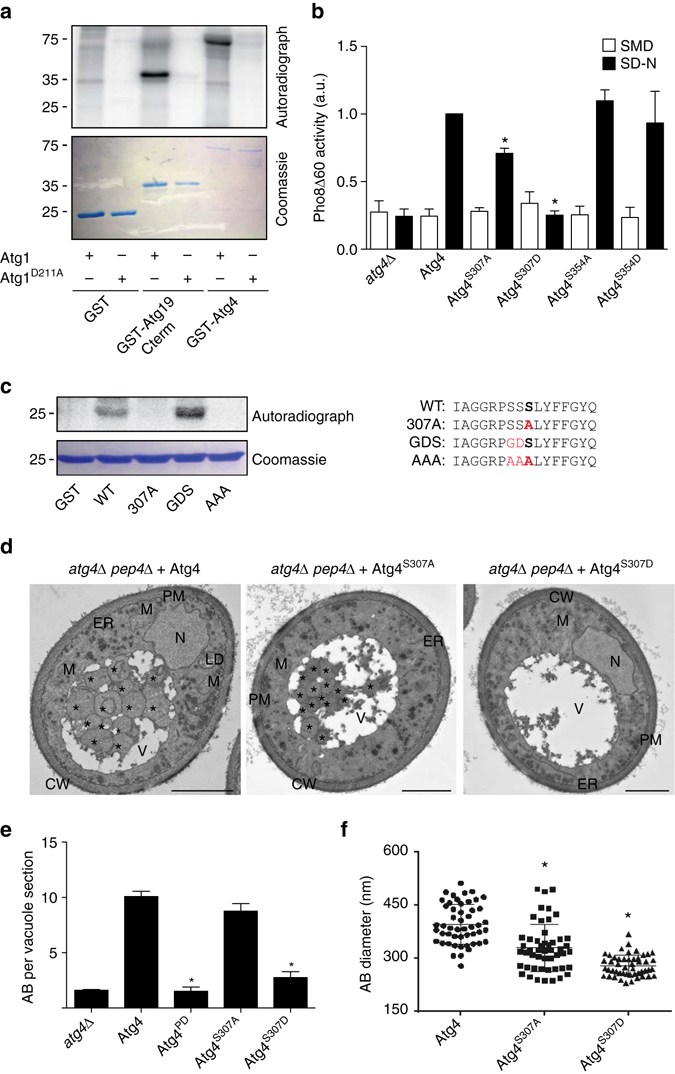
Atg1 phosphorylation of Atg4 inhibits autophagy. **a** GST, GST-Atg19Cterm, and GST-Atg4 were expressed in *E. coli*, immobilized on beads and in vitro phosphorylated with soluble Atg1-TAP and Atg1^D211A^-TAP complexes. The phosphorylation of the substrates was analyzed by autoradiography while their amounts were assessed by Coomassie brilliant blue staining of SDS-PAGE gels. **b** The *atg4Δ* (SAY130) strain carrying an empty pRS416 vector (*atg4Δ*) or plasmids expressing different Atg4 variants (Atg4, Atg4^S307A^, Atg4^S307D^, Atg4^S354A^, or Atg4^S354D^) was grown in SMD or nitrogen starved SD-N for 3 h before measuring Pho8Δ60 activity in cell lysates. The symbol * indicates statistical significance (*p* < 0.01) with the cells carrying Atg4 was calculated with the paired two-tailed Student’s *t*-test and arbitrary units (a.u.). **c** GST fusions of the indicated peptides (*right panel*) were expressed in *E. coli* and analyzed as in **a**. The amino acid in position 307 is in bold and mutated amino acids are in *red*. **d** The *atg4Δ pep4Δ* strain transformed with integration plasmids expressing Atg4-GFP (SAY144), Atg4^S307A^-GFP (JSY164), or Atg4^S307D^-GFP (JSY165) was grown in YPD to an early log phase and then nitrogen starved in SD-N for 3 h before processing the samples for EM. Autophagic bodies *AB* are highlighted in the EM micrographs with *asterisks*. *CW* cell wall; *ER* endoplasmic reticulum; *LD* lipid droplet; *M* mitochondrion; *N*, nucleus; *PM* plasma membrane; *V* vacuole. *Scale bar*, 1 µm. **e** Quantification of the average number of AB per vacuole section in the samples of **d**. Significant differences (*p* < 0.0001) between the various Atg4 mutants and the WT are indicated with the symbol * and were calculated with the paired two-tailed Student’s *t*-test. **f** Determination of the average diameter of the AB in WT, Atg4^S307A^, and Atg4^S307D^ samples of **d** (*n* = 50). Significant differences (*p* < 0.01) with the WT are indicated with the symbol * and were calculated with the paired two-tailed Student’s *t*-test

In our search for regulators of Atg4 activity, we made the assumption that this factor should possess two main characteristics, i.e., to be a protein present on autophagosomal membranes and be able to reversibly modify its substrates. The Atg1 kinase fits with this profile, because it dynamically localizes to the PAS^17^, and it has been shown that its phosphorylations can be antagonized by phosphatases^18^. Interestingly, the sequence analysis of Atg4 revealed that it contains at least seven putative Atg1 phosphorylation consensus sites (Supplementary Fig. 1a)^19^. We therefore decided to explore whether Atg4 is a substrate of Atg1. Purified Atg1 complexes containing either Atg1 or kinase-dead Atg1 (Atg1^D211A^) were incubated with [^32^P]-ATP and recombinant GST-Atg4, GST or the GST-tagged C-terminus of Atg19 (Atg19Cterm), a positive control for Atg1 phosphorylation^20^. The wild-type (WT) Atg1 complex, but not the one containing Atg1^D211A^, phosphorylated Atg4 and Atg19Cterm but not GST alone, indicating that Atg4 could indeed be a substrate of Atg1 kinase (Fig. 1a and Supplementary Fig. 6a).

To identify the residues involved in autophagy regulation, we individually mutated the putative phospho-acceptor serines in the different Atg1 phosphorylation consensus sites to alanine or aspartate to generate non-phosphorylable or phospho-mimicking forms of Atg4, respectively. The resulting Atg4 variants were expressed in an *atg4Δ* strain carrying Pho8Δ60 and autophagy progression was determined enzymatically^21^. Only cells expressing Atg4^S307D^ exhibited an autophagy block identical to the one in the *atg4Δ* mutant carrying an empty vector (Fig. 1b). Interestingly, the non-phosphorylable version of the same serine, i.e., Atg4^S307A^, could not completely bypass the autophagy impairment in *atg4Δ* cells (Fig. 1b). These phenotypes were not due to an effect of those mutations on the protein structure because introduction of a cysteine at the same position, did not impact autophagy (Supplementary Fig. 1b). Protein mass spectrometry analysis of Atg4^V297R,Q314K^-GFP isolated from WT cells revealed that the peptide containing serine 307 (S307) is indeed phosphorylated in vivo (Supplementary Fig. 2a and Supplementary Table 2). Atg4^V297R,Q314K^ is a version of Atg4 where two extra trypsin cleavage sites were introduced to obtain peptides containing the region of interest detectable by protein mass spectrometry (Supplementary Figs. 2b and 7b). Because of the very low abundance of the phospho-peptide of interest and the presence of 3 consecutive serines, however, we could not assign with certitude a phosphorylation to S307. To precisely determine the residue modified by Atg1, we performed an in vitro kinase phosphorylation assay. We confirmed that a peptide containing the Atg1 phosphorylation consensus region around serine 307 (WT) was phosphorylated by Atg1 like the positive control peptide, i.e., GDS (Fig. 1c and Supplementary Fig. 6b). As expected, the mutant peptides S307A and AAA were not phosphorylated (Fig. 1c and Supplementary Fig. 6b). We concluded that the S307 residue can be modified by Atg1.

To further assess the role of S307 in autophagy, we examined by electron microscopy the presence of autophagic bodies (AB) in cells lacking the major vacuolar protease Pep4^21^. The *atg4Δ pep4Δ* strain expressing Atg4^S307D^ exhibited the same severe decrease in the number of AB as the one carrying protease-dead Atg4 (Atg4^PD^)^10^ or an empty vector (*atg4Δ pep4Δ*) (Fig. 1d and Supplementary Fig. 3a). The very few AB observed in the vacuole lumen of this mutant have a strongly reduced size (Fig. 1d, e). In contrast, the strain expressing Atg4^S307A^ displayed the same number of AB as the control, i.e. cells carrying Atg4 (Fig. 1d, e). Interestingly, the average diameter of the AB in this mutant was smaller than in the control (Fig. 1f), also explaining its slightly lower autophagy activity (Fig. 1b) and impaired aminopeptidase 1 (Ape1) maturation (Supplementary Fig. 3b)^22^.

**Fig. 2:**
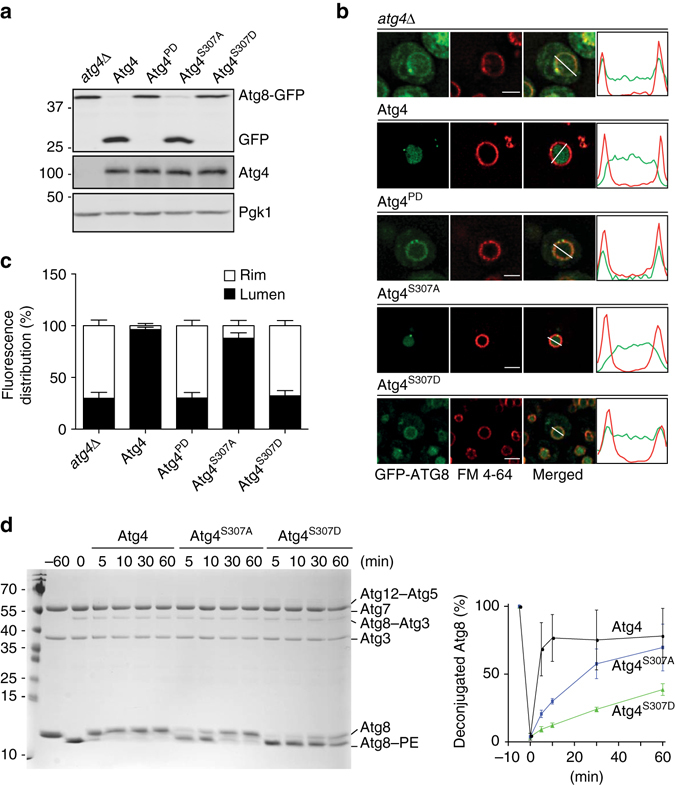
Atg4 phosphorylation on serine 307 blocks Atg4 function. **a** The *atg4Δ* cells carrying the integration plasmid pCuATG8GFP(403) (SAY114) and an empty vector (*atg4Δ*) or plasmids expressing the indicated 13xmyc-tagged Atg4 variants were grown in SMD and nitrogen starved in SD-N medium for 3 h. Proteins were precipitated with TCA and analyzed by western blot. **b** The atg4Δ strain carrying the integration plasmid pCuGFPAtg8ΔR(305) (JSY151) or plasmids expressing the indicated 13xmyc-tagged Atg4 variants were grown in SMD, nitrogen starved in SD-N for 3 h, labeled with the vacuole-specific dye FM 4-64 and imaged. Differential interference contrast (DIC). *Scale bar*, 5 µm. Fluorescence intensity plots of representative vacuoles are shown (indicated by *white lines*). **c** Quantification of GFP-Atg8∆R distribution on vacuole, i.e., rim vs. lumen, in cells imaged in **a** was performed as described in Methods. Results are mean ± SD (*n* = 50). **d** Atg8ΔR was conjugated to SUVs via the addition of Atg7, Atg3, Atg12–Atg5, MgCl_2_, and ATP. Deconjugation was initiated by the addition of WT Atg4 or the indicated mutants. Samples were collected after 5, 10, 30, and 60 min, and separated on a 11% SDS gel supplemented with 4.5 M urea. The *graph* on the *right* shows the quantification of three independent experiments

The similarity in phenotype shared by Atg4^S307D^ and Atg4^PD^ prompted us to analyze the proteolytic activity of the Atg4^S307D^ and Atg4^S307A^ mutants. First, we assessed the post-translational C-terminal cleavage of Atg8 by Atg4 using the Atg8-GFP chimera^8^. This analysis revealed that the Atg4^S307D^ mutant is proteolytically inactive similarly to Atg4^PD^ (Fig. 2a and Supplementary Fig. 7a). Atg4^S307A^, in contrast, behaved as WT Atg4 and normally cleaved Atg8-GFP (Fig. 2a and Supplementary Fig. 7a).

Expression of GFP-Atg8∆R in an *atg4∆* background allows the conjugation of Atg8 to PE independently of Atg4, permitting to specifically analyze the deconjugating capacity of Atg4^15^. As reported, PE-anchored GFP-Atg8 failed to be released from the surface of autophagosomes in *atg4∆* cells carrying either an empty vector or Atg4^PD^, and thus the fluorescence signal was mainly detected on the vacuolar limiting membrane highlighted with the specific vacuolar membrane dye FM4-64 (Fig. 2b, c)^15^. Complementation of the *atg4∆* mutant with WT Atg4 led to normal Atg8 recycling and delivery of autophagosomes into the vacuolar lumen (Fig. 2b, c)^15^. The same was observed in cells expressing Atg4^S307A^ indicating that GFP-Atg8–PE was normally processed upon autophagosome completion. In contrast, the GFP signal was mainly localized to the vacuolar rim in cells harboring Atg4^S307D^, revealing that this mutant is unable to recycle GFP-Atg8–PE, as observed in the Atg4^PD^ mutant (Fig. 2b, c). The fluorescence microscopy observations about the distribution of the GFP signal were confirmed by western blot analysis, in which higher amounts of free GFP were observed in WT Atg4 and Atg4^S307A^ strains, in which GFP signal was localized inside the vacuole, indicating normal autophagy flux (Supplementary Fig. 3b, c). To acquire more information about the dynamics of autophagosomes in presence of the different Atg4 variants, the same strains, which express GFP-Atg8∆R and thus allow to bypass the post-translational C-terminal cleavage defect of cells expressing Atg4^S307D^ (Fig. 2a), were examined by time-lapse microscopy. As shown in Supplementary Fig. 4 and Supplementary Movie 1, the average life time of autophagosomes in the strain carrying WT Atg4 was slightly longer than the one reported, i.e., 11.73 ± 0.96 min vs. 5–8 min^23^, probably because cells expressing the same GFP-Atg8∆R construct generate bigger autophagosomes^15^. The rate of formation and/or fusion of autophagosomes in cells expressing Atg4^S307A^ was longer (20.01 ± 1.08 min) and these vesicles appeared to have more GFP-Atg8 (Supplementary Fig. 4 and Supplementary Movie 2). These alterations possibly lead to a reduction in the autophagic flux providing a possible explanation for its slightly lower autophagic activity (Fig. 1b) and impaired Ape1 maturation (Supplementary Fig. 3b) of the Atg4^S307A^ strains. The autophagosome life time in the Atg4^S307D^ mutant (8.59 ± 2.14 min) did not significantly differ from that in the WT (Supplementary Fig. 4 and Supplementary Movie 3). However, the strongly reduced size of these carriers (Fig. 1d, e) implies that the time of autophagosome formation in Atg4^S307D^-expressing cells is a fraction of the one in the WT strain resulting in a marked reduction of the fusion events.

To further demonstrate that phosphorylation of S307 modulates Atg4-mediated deconjugation of Atg8 from PE, we examined the functionality of the different Atg4 mutants in vitro. Atg8–PE was completely deconjugated from small unilamellar vesicles (SUVs) 10 min after the addition of Atg4 (Fig. 2d). The deconjugation of Atg8 from PE by Atg4^S307A^ was slightly delayed compared to WT Atg4. In agreement with the in vivo assay, Atg4^S307D^ displayed a very severe defect in Atg8–PE processing (Fig. 2d).

### Atg4 phosphorylation affects its interaction with Atg8

**Fig. 3:**
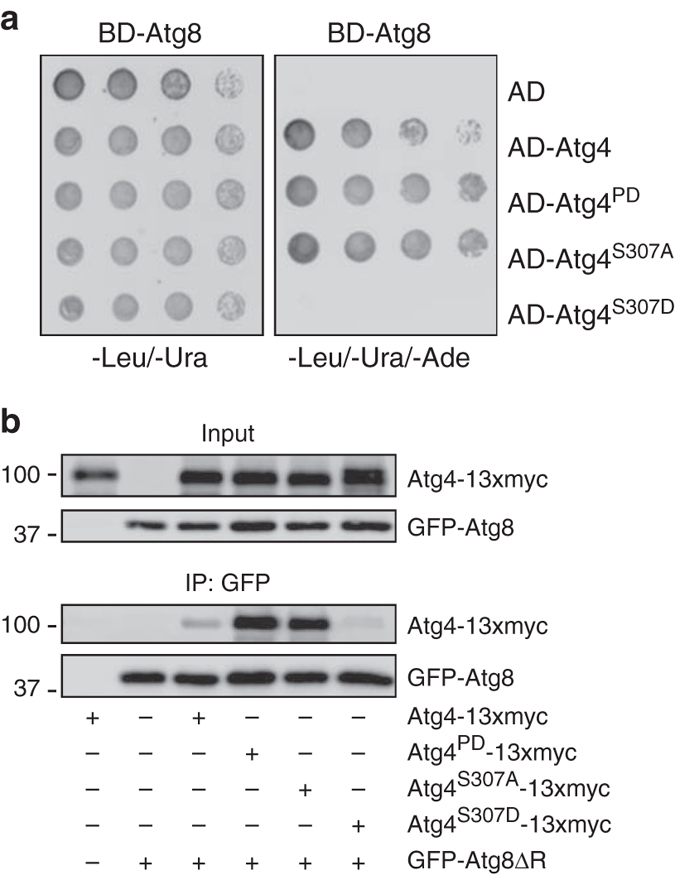
The phosphorylation state of S307 modulates Atg4 interaction with Atg8. **a** Atg4 and Atg8 were fused to the activation domain (AD) and/or the DNA-binding domain (BD) of Gal4, respectively. Plasmids were transformed into the PJ69-4A strain and colonies were spotted on medium lacking leucine and uracil (control plate) or leucine, uracil, and adenine (test plate). Growth on the test plate indicates interaction. The control was cells expressing BD-Atg8 alone. **b** Phosphorylation of S307 blocks the interaction of Atg4 with conjugated Atg8. The *atg4Δ* (SAY084) mutant transformed with WT Atg4-13xmyc and the *atg4Δ* strain with stably integrated pCuGFPAtg8ΔR(305) (JSY151) carrying a plasmid expressing the 13xmyc-tagged WT or the indicated Atg4 variants, were exponentially grown and nitrogen starved in SD-N for 1 h. Cell lysates were subjected to pull-down experiments using GFP-trap sepharose beads. Isolated proteins, 4% of cell lysate (input) or 100% of the pull-down material (IP), were resolved by SDS-PAGE and analyzed by western blot using anti-myc and anti-Atg8 antibodies

Atg1 phosphorylation at S307 could inhibit Atg4 functionality by either allosterically altering the catalytic site or by interfering with the substrate binding. We first tested the Atg4–Atg8 interaction using the yeast two-hybrid (Y2H) assay. As shown in Fig. 3a, cells exclusively expressing Atg8 did not grow on selective medium whereas those expressing both Atg4 and Atg8 displayed cell growth, confirming the interaction between these two proteins (Fig. 3a)^24^. Noteworthy, the interaction of Atg4^S307A^ and Atg4^PD^ with Atg8 was stronger than the one detected between Atg4 and Atg8. In contrast, cells carrying Atg4^S307D^ and Atg8 did not grow on the selective medium implying that this Atg4 mutant is unable to interact with Atg8 (Fig. 3a). The Y2H assay, however, cannot distinguish between Atg4 interaction with Atg8 or Atg8–PE. To overcome this limitation and test the binding of Atg4 variants to Atg8–PE, we performed a pull-down experiment in cells expressing GFP-Atg8ΔR. In line with the Y2H results, Atg4^PD^ and Atg4S^307A^ interaction with Atg8–PE was considerably stronger than the one observed with WT Atg4 (Fig. 3b and Supplementary Fig. 8). Atg4^S307D^ showed scarce binding to Atg8–PE indicating that this mutant lost almost completely its ability to interact with both Atg8 and Atg8–PE (Fig. 3b and Supplementary Fig. 8). Taken together, these data suggest that phosphorylation at S307 modulates the binding of Atg4 to Atg8.

### Atg1 and Atg4 interact on autophagosomal membranes

**Fig. 4:**
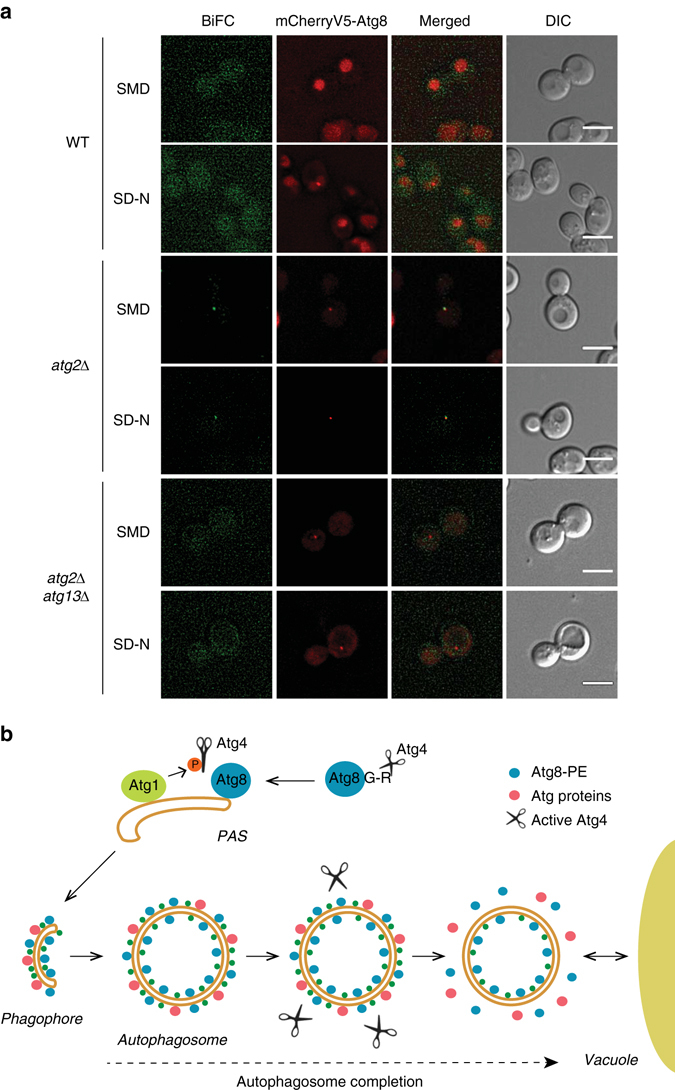
Atg1 and Atg4 interact at the PAS. **a** Atg1–Atg4 interaction at the PAS was visualized by BiFC. WT (JSY185), *atg2Δ* (JSY190), and *atg2Δ atg13Δ* (JSY215) cells expressing both endogenous Atg1–VN and Atg4–VC, and carrying a pCumCherryV5ATG8 plasmid were grown in SMD before being nitrogen starved in SD-N medium for 1 h. Fluorescence images were taken before and after nitrogen starvation. Differential interference contrast (DIC). *Scale bar*, 5 µm. **b** Mechanistic model for the regulation of Atg4 during autophagy. Newly synthesized Atg8 is constitutively processed by the cysteine protease Atg4 in the cytoplasm, where it is not inhibited by Atg1. After the cleavage of the C-terminal arginine, a glycine residue is exposed allowing Atg8 to be conjugated to the PE on autophagosomal membranes at the PAS. During the phagophore expansion, the Atg4 adjacent or coming in proximity of autophagosomal membranes, is locally inhibited by the action of the Atg1 kinase complex. Upon autophagosome completion, the release of Atg1 form autophagosomal membranes and/or its local inactivation allows Atg4 to act on Atg8–PE and release Atg8 from the PE anchor. This molecular mechanism, possibly together with other unknown regulatory events, would drive the dissociation of other Atg proteins from the surface of autophagosomes allowing their subsequent fusion with vacuoles

Atg4 phosphorylation at S307 blocks Atg8 proteolytic processing and recycling. Hence, Atg4 must be spatially regulated to allow the initial cleavage of Atg8 essential for its conjugation to PE and also avoid a premature Atg8–PE deconjugation until autophagosome biogenesis is completed. With Atg1 being a key regulator at the PAS and Atg4 one of its substrates (Fig. 1a, c), their interaction on autophagosomal membranes would provide a local regulation of Atg4 function. To address this notion, we performed a bimolecular fluorescence complementation (BiFC) assay^25^. In strains expressing only the N (VN) or the C-terminal (VC) fragment of Venus fused with Atg1 and Atg4, respectively, no fluorescence signal was detected (Supplementary Fig. 3d). BiFC signal was also absent in the strain carrying both Atg4–VC and Atg1–VN (Fig. 4a), probably due to the transient location of Atg proteins at the PAS. In agreement with this, a perivacuolar punctuate BiFC signal became evident in an *atg2∆* mutant in which Atg1 associates more pronouncedly to the PAS^17^. These BiFC puncta co-localized with mCherryV5-Atg8, supporting that Atg4 interacts with Atg1 at this specific location (Fig. 4a). Additional deletion of *ATG13* in this background prevents recruitment of Atg1 to the PAS^17^ and led to the disappearance of BiFC signals confirming that the interaction between Atg1 and Atg4 specifically takes place on autophagosomal membranes (Fig. 4a).

## Discussion

The recruitment of Atg proteins to the PAS and their dissociation from the surface of complete autophagosomes must be subjected to spatial and temporal regulation to avoid the formation of aberrant intermediate structures. It has been shown that Atg4 constitutively deconjugates Atg8 from PE on all membranes except the PAS, suggesting the existence of regulatory elements protecting Atg8–PE from cleavage by Atg4 at this site^9^. Here, we found that active Atg1 inhibits Atg4 action at the PAS. In contrast to the rest of the Atg proteins^17^, Atg4 does not localize at this location suggesting that it only very transiently associates with it^9^. As a result, a possible mechanistic model is that Atg1 prevents Atg4 interaction with its substrate at the PAS, i.e., Atg8–PE. This transient inhibition could be one of the mechanisms that allow the synthesis and the maintenance of the Atg8–PE pool required for the formation of an autophagosome. At the completion of an autophagosome, inactivation of Atg1 would permit Atg4 to act on the Atg8–PE on autophagosomal membranes (Fig. 4b)^11, 13, 15^. This hypothetical model is indirectly supported by the observation that the human homologue of Atg13, very likely in complex with Atg1/ULK1, is one of the first Atg proteins dissociating from autophagosomes when those separate from omegasomes, a step that possibly takes place at their sealing^26^.

Atg1 directly inhibits Atg4 protease activity through phosphorylation of S307. The lack of interaction between Atg4^S307D^ and Atg8 (Fig. 3) indicates that this modification affects Atg4 binding to its substrate rather than negatively regulating its proteolytic activity by altering the catalytic site. This is indirectly supported by the observation that catalytically dead Atg4^PD^ binds Atg8 (Fig. 3). Nonetheless, based on the predicted Atg4 structure (Supplementary Fig. 5a), S307 is close to amino acids D322 and H324, which correspond to D278 and H280 in ATG4B’s catalytic site^27^. In particular, S307 faces H324 and when phosphorylated, its interaction with the positively charged H324 could induce a conformational change that alters the predicted Atg8-binding region (Supplementary Fig. 5a). The interaction between phosphorylated S307 and H324, however, could also lead to a closure of the catalytic pocket. Our multiple approaches to quantify the degree of phosphorylation of S307 in vivo have been unsuccessful probably due to the very low amounts of this modification, which make it not always detectable. This suggests that only a very small subpopulation of S307 is modified and/or is a very transient phosphorylation of S307. One possibility is that Atg4 approaching the Atg8–PE on autophagosomal membranes gets phosphorylated and, it is rapidly dephosphorylated after immediate release into the cytoplasm. Another possible scenario emerges from few recent publications. A structural study describing the mechanism of *Legionella* RavZ-mediated LC3–PE C-terminal processing reached the conclusion that the N-terminal and C-terminal LIR motifs in RavZ are essential to bind the substrate simultaneously and open the catalytic groove to allow the access to the bond that has to be cleaved^28^. LIR motifs, plus a newly identified domain, are important for the recognition and deconjugation of lipidated Atg8/LC3 proteins by Atg4 proteases in yeast and mammals^29, 30^. It is thus plausible that regulation of LIR motif-mediated recruitment to autophagosomal membrane is the major mechanism for Atg8–PE processing, and Atg1 kinase inhibition represents an extra control mechanism. This latter would operate when the catalytic groove opens and therefore S307 could be of easily access for Atg1.

Alignment of the amino acid sequence of Atg4 protein family members from different organisms shows that Atg4 proteins have either a serine or an alanine in the position corresponding to that of yeast S307 (Supplementary Fig. 5b). The phylogenetic analysis reveals an evolutionary relation within Atg4 proteins with serines and within those with alanines (Supplementary Fig. 5c). The higher Atg8–PE deconjugating activity of the Atg4 isoforms with an alanine compared to those with a serine^31, 32^ suggests that they might be modulated by a different and very likely, less stringent mechanism. Human ATG4B is also inhibited by ULK1 (the mammalian homologue of Atg1) through phosphorylation at S316^33^. The corresponding serine in yeast, i.e., S354, however, shows no major defect in autophagy progression when mutated to aspartate or alanine (Fig. 1b). Interestingly, the residue equivalent to S307 of yeast Atg4 is substituted by an alanine in ATG4B (Supplementary Fig. 5b). Altogether these observations reveal that although phosphorylation by Atg1 homologues is an evolutionary conserved mechanism to negatively regulate Atg4, different serine residues are used. Future studies are needed to decipher the significance of this difference.

## Methods

### Plasmids

The pATG4GFP(416) plasmid was generated by PCR amplification of *ATG4* promoter and the *ATG4*-*GFP* fusion from the MNY006 strain genome, and its subsequent cloning into the pRS416 vector^34^ as a *Kpn*I/*Asc*I fragment. The pATG413xmyc(416) plasmid was obtained by replacing *GFP* in pATG4GFP(416) with the sequence for the 13xmyc tag from the pFA6a13xmycTRP1 plasmid^35^ using *Pac*I and *Asc*I. Protease-dead (C147S), the cysteine mutant (S307C) and phospho-mutant versions of Atg4 were created by inserting nucleotide changes in the pATG4GFP(416) and pATG413xmyc(416) plasmids using the site-direct mutagenesis kit (Stratagene, LaJolla, CA). The correct introduction of the point mutations was verified by DNA sequencing. The pRS416 plasmid backbone was replaced with the one of the pRS406^34^ using *Kpn*I and *Sac*I, to create the vectors integrating the various *ATG4-GFP* constructs into the genome. The integrative vectors expressing untagged Atg4, Atg4^S307A^ and Atg4^S307D^ under the control of their own promotor (i.e, pATG4(406), pATG4^S307A^(406), and pATG4^S307D^(406) were obtained by cloning PCR products from the appropriate template plasmids as *Kpn*I-*Asc*I fragments into pATG4GFP(406), a strategy that allows to remove *GFP*.

The pTEFATG4^V297R,Q314K^-GFP(416) plasmid was generated by triple ligation into the pRS416 vector digested with *Kpn*I and *Xma*I, of a fragments carrying the *TEF1* promoter (digested with *Kpn*I and *Pac*I) and the Atg4^V297R,Q314K^ ORF (digested with *Pac*I and *Xma*I). The sequence coding for Atg4^V297R,Q314K^ was obtained beforehand by site-directed mutagenesis of the pATG4GFP(416) construct. These two point mutations, which do not affect the functionality of Atg4 (Supplementary Fig. 2b), were introduced to add new trypsination sites in order to generate a peptide of the region of interest detectable by protein mass spectrometry analysis.

The pCuGFPATG8ΔR(305) and pCuATG8GFP(403) plasmids were generated by replacing the vector backbone of the pCuGFPATG8ΔR(406)^15^ and pAUT7GFP(416) plasmid^8^ with the one from the pRS305 and pRS403 vectors^34^, respectively, using *Sac*I and *Xho*I.

The yeast two-hybrid plasmids were created by amplifying the ORF of *ATG4* and its variants by PCR from the pATG413xmyc plasmids described above, and cloning them as a *XmaI/SalI* fragment into the pGAD-C1 vector^36^. The pGBDU-Atg8 plasmid was described elsewhere^37^.

To create the plasmids expressing GST-tagged Atg4 peptides, complementary primers encoding for the peptides and containing the overhanging ends generating *EcoR*I and *Xho*I restriction sites, were annealed together to create the insert and cloned into pGEX4T3 vector as *EcoR*I*/Xho*I fragments.

The pCumCherryV5ATG8 construct has been described elsewhere^38^.

### Yeast strains

The Saccharomyces *cerevisiae* strains used in this study are listed in Supplementary Table 1. For gene disruptions, the coding regions were replaced with *K. lactis URA3* or *LEU2*, the *S. cerevisiae TRP1*, the *Schizosaccharomyces pombe HIS5* or the *Klebsiella pneumoniae hphNT1*gene by homologous recombination using PCR products generated with primers containing 60 bases of identity to the regions flanking the open reading frames^35, 39, 40^. Chromosomal tagging of the *ATG1* or *ATG4* genes at the 3’end was done by PCR-based integration of the N-terminus or the C-terminus of the Venus tag using pFA6a-VN-His3MX6 and pFA6a-VC-TRP1 as template plasmids, respectively^25^. PCR verification, western blotting using antibodies recognizing the tags and analysis of Ape1 processing were used to confirm all deletions and integrations as well as the functionality of all the protein fusions.

### Media

Yeast cells were grown in rich (YPD; 1% yeast extract, 2% peptone, and 2% glucose) or synthetic minimal (SMD; 0.67% yeast nitrogen base, 2% glucose, and amino acids and vitamins as needed) medium. Autophagy was induced by transferring cells into a nitrogen starvation medium (SD-N; 0.17% yeast nitrogen base without amino acids and ammonium sulfate, and 2% glucose) during 1 and 3 h.

### Antibodies and rReagents

Primary antibodies or sera for protein or tag detection were: anti-GFP antibody (Roche, cat# 11814460001, 1:3000 dilution), anti-myc antibody (Santa Cruz, cat# sc-40, 1:10 000 dilution), anti-Ape1 antibody (1:3000 dilution)^38^ and anti-Atg8 (1:5000 dilution)^30^. The anti-Pgk1 antiserum was generated by immunization of New Zealand White rabbits with the CLKYFGKALENPTR peptide (New England Peptides) and used at a 1:1000 dilution. Alexa-680 conjugated anti-rabbit or mouse IgG antibodies were used as secondary antibodies (Life technologies, 1:5000 dilution).

### Fluorescence microscopy

Fluorescence signals were visualized with a DeltaVision RT fluorescence microscope (Applied Precision) equipped with a CoolSNAP HQ camera (Photometrix). Images were generated by collecting a stack of 20 pictures with focal planes 0.2 μm apart in order to cover the entire volume of a yeast cell and by successively deconvolving them using the SoftWoRx software (Applied Precision). A single-focal plane is shown at each time point. The FM 4–64 dye (Invitrogen) was used to specifically stain the vacuolar rim^41^. The GFP intensity in vacuolar rim or lumen was determine as an average of the intensity of several points in each region by using the multi-point tool in ImageJ.

For time-lapse imaging experiments, cells nitrogen starved in SD-N medium and stained with the CellTracker Blue 7-amino-4-chloromethylcoumarin (CMAC) dye (Invitrogen) for 30 min, were imaged every 30 s, collecting a Z-stack of six pictures with focal planes 0.30 μm apart. Images were deconvolved and mounted into movies before measuring the life of GFP-Atg8∆R using the SoftWoRx software. The time point at which GFP-Atg8∆R appeared as a punctuate structure was considered as time 0 min. 2D projections of Z-stack images were employ to quantify the relative intensity of GFP-Atg8∆R-positive structures as previously described^23^ and setting to 1 the relative intensity of cells expressing WT Atg4.

### Yeast two-hybrid assay

The plasmids pGBDU-C1 and pGAD-C1 carrying *ATG8* and *ATG4* or its mutated forms, respectively, were transformed into the PJ69-4A test strain and grown on SMD medium lacking leucine and uracil^36^. Colonies were then spotted on SMD medium lacking leucine and uracil (control plate) or leucine, uracil and adenine (test plate).

### Immunoprecipitations

The equivalent of 100 OD_600_ growing cells was transferred to SD-N medium for 1 h, harvested by centrifugation and resuspended in 1 ml of lysis buffer (45 mM HEPES, pH 7.4, 150 mM NaCl, 1 mM EDTA, 10% glycerol, 0.5% Tween-20) supplemented with 1 mM PMSF, Complete protease inhibitors (Roche), 10 mM β-glycerophosphate, 50 mM NaF, and 1 mM Na_3_VO_4_. After addition of glass beads, cells were lysed by vortexing at 4 °C for 15 min and lysates cleared by centrifugation at 15 000 g for 5 min at 4 °C. The supernatant was incubated with 25 µl of pre-washed GFP-trap sepharose beads (ChromoTek) on a rotating wheel for 1.5 h at 4 °C. Beads were finally washed 3 times in 1 ml of lysis buffer, resuspended in loading buffer and analyzed by SDS-PAGE followed by western blot.

### Electron microscopy

Fifteen OD_600_ unit equivalents of cells were resuspended in 1 ml of freshly prepared ice-cold 1.5% KMnO4 (Sigma) and transferred into a 1.5 ml microfuge tube. After topping up the tube with 0.5 ml of the same solution to exclude air, samples were mixed on a rotatory wheel for 30 min at 4 °C. This operation was repeated once more before washing the pellets five times with 1 ml of distilled water. Cells were then dehydrated in increasing amounts of acetone (10, 30, 50, 70, 90, 95, and three times 100%) by incubation on a rotatory wheel for at least 20 min at room temperature, at each step. After centrifugation, pellets were resuspended in 33% Spurr’s resin in acetone and mixed on the same device for 1 h at room temperature. This operation was repeated twice overnight and successively during all the day in 100% Spurr’s resin. The Spurr’s resin mixture was prepared by mixing 10 g of 4-vinylcyclohexene dioxide (or ERL4206), 4 g of epichlorohydrin-polyglycol epoxy resin 736, 26 g of (2-nonen-1-yl)succinic anhydride, and 0.4 g of N,Ndiethylethanolamine (all from Sigma). Incubating the preparations overnight at 70 °C polymerized the Spurr’s resin. Section of about 65–80 nm were then cut using an Ultracut E ultramicrotome (Leica Microsystems) and transferred on Formvar carbon-coated copper grids. Sections where then stained first with 6% uranyl acetate for 30 min at room temperature and then with a lead-citrate solution (80 mM lead nitrate, 120 mM sodium citrate, pH 12) for 2 min before being viewed^42^. To determine the number of AB per vacuole and their diameter, three different grids with sections obtained from the same preparation were evaluated. For every grid, the number and diameter of AB in 50 cells with apparent vacuoles was determined. Error bars represent the standard deviation from the counting of the three grids.

### Protein expression and purification

Atg3, Atg4, Atg4 mutants, Atg7, Atg8, and the Atg12–Atg5 complex were expressed and purified *E. coli* Rosetta pLySS cells as described.

Full length Atg3 and Atg19Cterm were expressed as N-terminal GST fusion proteins from pGEX4T1^43^. Cells were grown at 37 °C to an OD_600_ of 0.8, induced with 50 μM isopropyl β-D-1-thiogalactopyranoside (IPTG), and grown for a further 16 h at 18 °C. Cells were pelleted and resuspended in a buffer containing 50 mM HEPES, pH 7.5, 300 mM NaCl, 2 mM MgCl_2_, 1 mM DTT and Complete protease inhibitors (Roche) and DNAse I (Sigma). Cells were lysed by freeze thawing and the lysate was centrifuged at 40 000 rpm (Beckman Ti45 rotor) for 40 min at 4 °C. The supernatant was incubated with glutathione beads (GE Healthcare) for 2 h at 4 °C. Beads were washed five times with 50 mM HEPES, pH 7.5, 300 mM NaCl, and 1 mM DTT, followed by two washes with 50 mM HEPES, pH 7.5, 1000 mM NaCl, 1 mM DTT and two washes with 50 mM HEPES, pH 7.5, 300 mM NaCl, and 1 mM DTT. The proteins were cleaved off from the GST tag by incubation with thrombin protease (Serva) overnight at 4 °C. The supernatant containing the cleaved off Atg3 and Atg19Cterm were diluted to reach a final salt concentration of 150 mM NaCl and further purified using a 16/60 Q-Sepharose column. The protein was eluted on a gradient ranging from 150 mM–1 M NaCl. Fractions containing Atg3 and Atg19Cterm were pooled, concentrated, and run on a 16/60 S75 size exclusion column in 50 mM HEPES, pH 7.5, 150 mM NaCl and 1 mM DTT.

Full length Atg7 was expressed as an N-terminal 6xhistidine-tagged protein from pOPTHrsTEV^43^. Cells were grown at 37 °C to an OD_600_ of 0.8, induced with 50 μM IPTG, and grown for a further 16 h at 18 °C. Cells were pelleted and resuspended in a buffer containing 50 mM HEPES, pH 7.5, 300 mM NaCl, 10 mM imidazole, 1 mM MgCl_2_, 2 mM β-mercaptoethanol, Complete protease inhibitors (Roche), and DNAse I (Sigma). Cells were lysed by freeze thawing and the lysate was centrifuged at 40 000 rpm (Beckman Ti45 rotor) for 40 min at 4 °C. The supernatant was incubated with nickel beads (5 Prime) for 2 h at 4 °C. Beads were washed with 50 mM HEPES, pH 7.5, 300 mM NaCl, 10 mM imidazole, and 2 mM β-mercaptoethanol, and the 6xhistidine-tag was cleaved off with TEV protease at room temperature. The Atg7 protein was diluted to reach a final salt concentration of 150 mM and further purified on 16/60 Q-Sepharose column. The protein was eluted using a gradient reaching from 150 mM–1 M NaCl. Fractions containing Atg7 were pooled, concentrated and run on a 16/60 S200 size exclusion column in 50 mM HEPES pH 7.5, 15 0 mM NaCl, and 1 mM DTT.

Atg8 lacking the C-terminal arginine (R117, Atg8∆R) was expressed as N-terminal 6xhistidine-tagged protein from pOPC-His-TEV-Atg8^43^. Cells were grown at 37 °C to an OD_600_ of 0.8, induced with 500 μM IPTG, and grown for a further 3 h at 37 °C. Cells were pelleted and resuspended in a buffer containing 50 mM HEPES, pH 7.5, 300 mM NaCl, 10 mM imidazole, 1 mM MgCl_2_, 2 mM β−mercaptoethanol, Complete protease inhibitors (Roche), and DNAse I (Sigma). Cells were lysed by freeze thawing and the lysate was centrifuged at 40000 rpm (Beckman Ti45 rotor) for 40 min at 4 °C. The supernatant was incubated with nickel beads (5 Prime) for 2 h at 4 °C. Beads were washed with 50 mM HEPES, pH 7.5, 300 mM NaCl, 10 mM imidazole and 2 mM β−mercaptoethanol, and the 10xhistidine-tag was cleaved off for several hours with TEV protease at room temperature. The Atg8 protein was diluted to reach final salt concentration of 150 mM and the protein was further purified on 16/60 SP-Sepharose column. The protein was eluted using a gradient reaching from 150–1000 mM NaCl. Fractions containing Atg8 were pooled, concentrated, and run on a 16/60 Superdex S75 size exclusion column in 50 mM HEPES pH 7.5, 150 mM NaCl, and 1 mM DTT.

The Atg5–Atg12 conjugate was produced by co-expression of 6xhistidine-tagged Atg5, Atg12, Atg10, and Atg7^43^. Cells were grown at 37 °C to an OD_600_ of 0.8, induced with 1 mM IPTG and grown for another 4 h at 37 °C. Collected cells were resuspended in the resuspension buffer (300 mM NaCl, 5 0 mM HEPES, pH 7.5, 10 mM imidazole, 2.5 mM Pefablock (Roth), 1 mM MgCl_2_, 2 mM β-mercaptoethanol, and DNAse I (Sigma), and disrupted by freeze-thaw method and sonication. The cleared lysate was applied to a HisTrap column (GE Healthcare) and the proteins were eluted by a step-wise imidazole gradient. The Atg5–Atg12 the eluate was concentrated using Amicon Ultra centrifugal filter (MW cut-off 30 kDa) and further purified using a 16/60 S200 size exclusion column (GE Healthcare). The protein complex was eluted from the column with 150 mM NaCl, 50 mM HEPES, pH 7.5, and 1 mM DTT.

Atg4 and its mutant versions were expressed as an N-terminal GST fusion proteins. Cells were grown at 37 °C until an OD_600_ of 0.8. Protein expression was induced by addition of IPTG to a final concentration of 0.1 mM and the protein was expressed at 18 °C overnight. The cell pellets were resuspended in 50 mM HEPES/KOH, pH 7.5, 300 mM NaCl, 1 mM DTT, 1 mM MgCl_2_, DNase I, Complete protease inhibitors (Roche), and PEFABLOC. Cells were lysed by freezing in liquid nitrogen and thawing, followed by brief sonication. The lysate was cleared by centrifugation at 200 000×*g* for 40 min at 4 °C and the supernatant was incubated with equilibrated glutathione beads (GE Healthcare) for 2 h at 4 °C. The beads were washed five times with 50 mM HEPES/KOH, pH 7.5, 300 mM NaCl, 1 mM DTT, two times with 50 mM HEPES, pH 7.5, 700 mM NaCl, 1 mM DTT, and finally two times with 50 mM HEPES, pH 7.5, 300 mM NaCl, 1 mM DTT. The protein was eluted with 50 mM HEPES, pH 7.5, 300 mM NaCl, and 1 mM DTT supplemented with 20 mM L-glutathione and cleaved with thrombin. The cleaved protein was run on a Superdex 200 16/600 column (GE Healthcare) and the peak fractions containing Atg4 were pooled, concentrated, and flash frozen in liquid nitrogen.

### Preparation of small unilamellar vesicles

SUVs used for the conjugation and deconjugation assays were composed of 65% DOPC, 30% DOPE and 5% PI (all purchased from Avanti Polar Lipids). 100 μl of the lipid stock (10 mg/ml) were transferred into a glass vial and dried under an argon stream. The dried lipids were dried additionally for 1 h in a desiccator. Subsequently, the dried lipids were subsequently incubated with SUV buffer (25 mM HEPES, pH 7.5, 137 mM NaCl, 2.7 mM KCl, 1 mM DTT) for 15 min. The lipids were resuspended by tapping and gently sonicated for 2 min in a water bath sonicator. The resuspended SUVs were then extruded 21 times through a 0.4 μm membrane followed by extrusion through a 0.1 μm membrane (Whatman Nucleopore, St Louis, MO) using the Mini Extruder (Avanti Polar Lipids). The final SUVs suspension has a concentration of 1 mg lipids/ml buffer.

### Atg8 conjugation and deconjugation assay using SUVs

The conjugation and deconjugation reactions were performed at 30 °C and all buffers, solutions and the SUVs with the exception of the proteins were pre-warmed to this temperature. Atg3 and Atg7 were used at a final concentration of 1 µM, whereas Atg12–Atg5 and Atg8∆R were used at a final concentration of 0.5 µM and 5 µM, respectively. ATP was added to a final concentration of 50 µM while MgCl_2_ was used at 1 mM. Conjugation reactions were stopped by the addition of 1000 units of calf intestinal phosphatase (New England Biolabs). For the deconjugation reaction, Atg4 was used at a final concentration of 25 nM. The reactions were then stopped by the addition of SDS-PAGE loading buffer and samples separated on 11% SDS-PAGE gels containing 4.5 M urea in the separating part.

### Protein mass spectrometry analysis

700 ml of late stationary grown *atg4Δ* cells (SAY084) transformed with the pTEFATG4^V297R,Q314K^-GFP(416) plasmid and nitrogen starved in SD-N medium for 1 h were lysed by cryogenic grinding in 45 mM HEPES, pH 7.4, 150 mM NaCl, 1 mM EDTA, 10% glycerol, 0.5% Tween-20 supplemented with 1 mM PMSF, Complete protease inhibitors (Roche), 10 mM β-glycerophosphate, 10 mM NaF, and 1 mM Na_3_VO_4_ buffer. Lysates were then cleared by centrifugation and incubated with 100 µl GFP-sepharose beads. Immuno-isolated Atg4^V297R,Q314K^-GFP was eluted in sample buffer and separated by SDS-PAGE before cutting the gel band containing the fusion protein. After in-gel trypsination, the resulting peptides were separated and analyzed by liquid chromatography-mass spectrometry (LC-MS) on a Q-Exactive plus MS instrument with an Ultimate 3000 nano RSLC LC system (Thermo). Peptides were fragmented by collision-induced dissociation, and the resulting peptide MS/MS spectra were used for identification of proteins and modifications, performed with the PEAKS software version 7.5 (Bioinformatics Solutions).

### In vitro Atg1 pPhosphorylation

Atg1-TAP and Atg1^D211A^-TAP, and the associated proteins, were immunoisolated from yeast grown in 2 l of YPD medium to an OD_600_ of 2 and treated with 220 nM rapamycin for 1 h, harvested by centrifugation, and washed in PBS, 2% glucose. Cells were then resuspended in the lysis buffer (PBS, 10% glycerol, 0.5% Tween-20, 1 mM NaF, 1 mM phenylmethylsulfonylfluoride, 1 mM Na_3_VO_4_, 20 mM β-glycerophosphate, protease inhibitor cocktail (Roche)) and frozen in droplets in liquid nitrogen. After cell disruption with a freezer mill (6770; SPEX), the extract was thawed in lysis buffer and cleared by centrifugation. The cleared extracts were then incubated with 160 μl of IgG-coupled magnetic beads (Dynabeads, Invitrogen) for 1 h at 4 °C with rotation. The beads were washed six times for 5–10 min in lysis buffer with rotation and cleaved in lysis buffer containing 0.5 mM DTT and the TEV protease for 1 h at 16 °C with slow shaking. The immunoisolated Atg1-TAP and Atg1^D211A^-TAP, and the associated proteins, were incubated in the phosphorylation mixture (10 μCi γATP, 25 mM MOPS pH 7.5, 1 mM EGTA, 10 mM Na_3_VO_4_, 15 mM MgCl_2_ total volume of 11 μl) with 2 µg of soluble GST, GST-Atg19Cterm, or GST-Atg4, purified from *E. coli* ^20^. After 20 min of incubation at 30 °C, the Atg1 bound beads were removed and the supernatant was assessed for radioactivity incorporation by phospho-imaging. GST-fused peptides were immobilized on GST beads and TEV eluted soluble Atg1 complexes were used for the in vitro phosphorylation reaction as described above.

### Pho8Δ60 assay

As previously described^21^, 5 OD_600_ equivalents of cells were lysed in 400 μl of ice-cold lysis buffer (20 mM PIPES, pH 6.8, 0.5% Triton X-100, 50 mM KCl, 100 mM potassium acetate, 10 mM MgCl_2_, 10 μM ZnSO_4_, 2 mM PMSF) by vortexing in presence of 100 µl of glass beads (0.4–0.6 mm in diameter) for 3–5 min at 4 °C. Lysates were centrifuged at 13000 g for 5 min at 4 °C. Then 100 μl of supernatant were mixed with 400 µl of ALP reaction buffer (250 mM Tris-HCl, pH 8.5, 0.4% Triton X-100, 10 mM MgCl_2_, 10 μM ZnSO_4_, 1.25 mM *p-*nitrophenyl phosphate) pre-warmed at 37 °C. Samples were incubated at 37 °C for 20 min before adding 500 μl of 1 M glycine, pH 11.0. After centrifuge at 13000 g for 2 min, the absorbance of the supernatant was measured at 400 nm. Enzymatic activity was calculated with the following formula 1000×OD_400_/(time×protein concentration in µg/ml) and expressed in arbitrary units.

### Western blot analyses

Western blot analyses were conducted as previously described^21^. Briefly, 2.5 OD_600_ equivalents of cells were collected by centrifugation at 13 000×*g* for 1 min and resuspended in 400 µl of ice-cold 10% trichloroacetic acid (TCA). After having left them on ice for at least 30 min, mixtures were centrifuged at 13000×*g* for 5 min at 4 °C and the protein pellets were resuspended in 1 ml of ice-cold acetone by sonication. Samples were subsequently put at −20 °C for at least 20 min before to be centrifuged at 13000×*g* for 5 min at 4 °C. Pellets were dried, resuspended in 80–100 µl of 1× Laemmli sample buffer (50 mM Tris-HCl, pH 6.8, 2% SDS, 10% glycerol, 1% β-mercaptoethanol) and boiled before to be loaded on SDS-PAGE gels. Proteins were finally transferred on PVDF membranes and detected using an Odyssey system (Li Cor Biosciences) and ImageJ software was used for processing of images and band quantification.

### Statistical analyses

Statistical analyses were done using the paired two-tailed Student’s *t*-test.

### Accession Numbers

All proteins used were from *Saccharomyces cerevisiae*. Atg3: NP_014404; Atg4: NP_014176.2; Atg5: NP_015176.1; Atg7: NP_012041.1; Atg8: NP_009475.1; Atg10: NP_013058.1; Atg12: NP_009776.1; Atg16: NP_013882.1.

### Data availability

All data generated or analyzed during this study are included in this published article and its Supplementary Information.

## Electronic supplementary material

Supplementary Movie 1

Supplementary Movie 2

Supplementary Movie 3

Supplementary Information
