# Supplementary material for: Atg4 proteolytic activity can be inhibited by Atg1 phosphorylation
Source: Nat Commun. 2017 Aug 18;8:295. doi: 10.1038/s41467-017-00302-3 (PMC5562703; doi:10.1038/s41467-017-00302-3)
Supplement: Supplementary file 4 — Supplementary Information [file 41467_2017_302_MOESM4_ESM.pdf]

Description of Supplementary Files

Title: Supplementary Information

Description: Supplementary Figures, Supplementary Tables, and Supplementary References

Title: Supplementary Movie 1
Description: Time-lapse imaging of GFP-Atg8.R puncta in Atg4 expressing cells.
The RGY306 strain was grown in YPD medium at 30˚C to a logarithmic phase before being nitrogen starved in SD-N medium for 30 min. Cells were also incubated with CMAC dye 10 min before image acquisition to visualize the vacuoles. The video were generated as described in the Methods section.

Title: Supplementary Movie 2
Description:Time-lapse imaging of GFP-Atg8.R puncta in Atg4S307A expressing cells.
The RGY297 strain was grown and imaged as in Supplementary Video 1.

Title: Supplementary Movie 3
Description: Time-lapse imaging of GFP-Atg8.R puncta in Atg4S307D expressing cells.
The RGY298 strain was grown and imaged as in Supplementary Video 1.

a

```
1   MQRWLQLWKM DLVQKVSHGV FEGSSEEPAA LMNHDYIVLG EVYPERDEES
51  GAEQCEQDCR YRGEAVSDGF LSSLFGREIS SYTKEFLLDV QSRVNFTYRT
101 RFVPIARAPD GPSPLSLNLL VRTNPISTIE DYIANPDCFN TDIGWGCMIR
151 TGQSLLGNAL QILHLGRDFR VNGNESLERE SKFVNWFNDT PEAPFSLHNF
201 VSAGTELSDK RPGEWFGPAA TARSIQSLIY GFPECGIDDC IVSVSSGDIY
251 ENEVEKVFAE NPNSRILFLL GVKLGINAVN ESYRESICGI LSSTQSVGIA
301 GGRPSSSLYF FGYQGNEFLH FDPHIPQPAV EDSFVESCHT SKFGKLQLSE
351 MDPSMLIGIL IKGEKDWQQW KLEVAESAII NVLAKRMDDF DVSCSMDDVE
401 VSSNSMKKDA SNNENLGVLE GDYVDIGAIF PHTTNTEDVD EYDCFQDIHC
451 KKQKIVVMGN THTVNANLTD YEVEGVLVEK ETVGIHSPID EKC
```

b

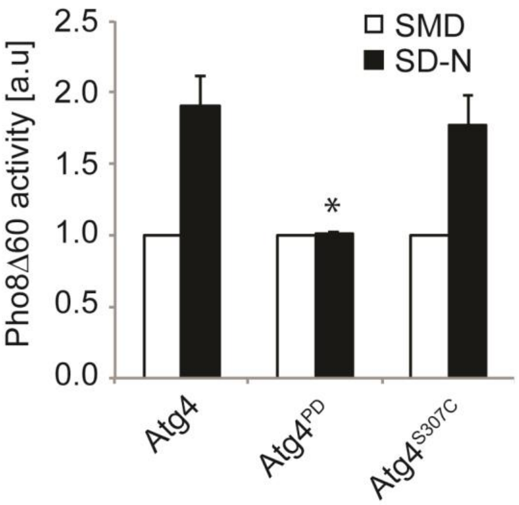

Supplementary Figure 1. Atg4 and amino acid sequence. (a) Atg4 contains 7 potential Atg1 phosphorylation sites. *Saccharomyces cerevisiae* Atg4 amino acid sequence. The potential phospho-acceptor serines in the Atg1 potential phosphorylation sites are in blue, S307 is in green and the residues that are putatively part of the catalytic site are in red.(b) The *atg4Δ* (SAY130) strain carrying a plasmid expressing Atg4, Atg4^{PD} or Atg4^{S307C} was grown in

SMD or nitrogen starved (SD-N) for 3 h before measuring Pho8Δ60 activity in cell lysates. Pho8Δ60 activity was expressed in and a.u. stands for arbitrary units and relative to the control SMD conditions. Error bars represent the SD of 5 independent experiments. The symbol * indicate statistical significance ($p<0.001$) with the cells carrying Atg4 and was calculated with the paired two-tailed Student's *t*'test.

a

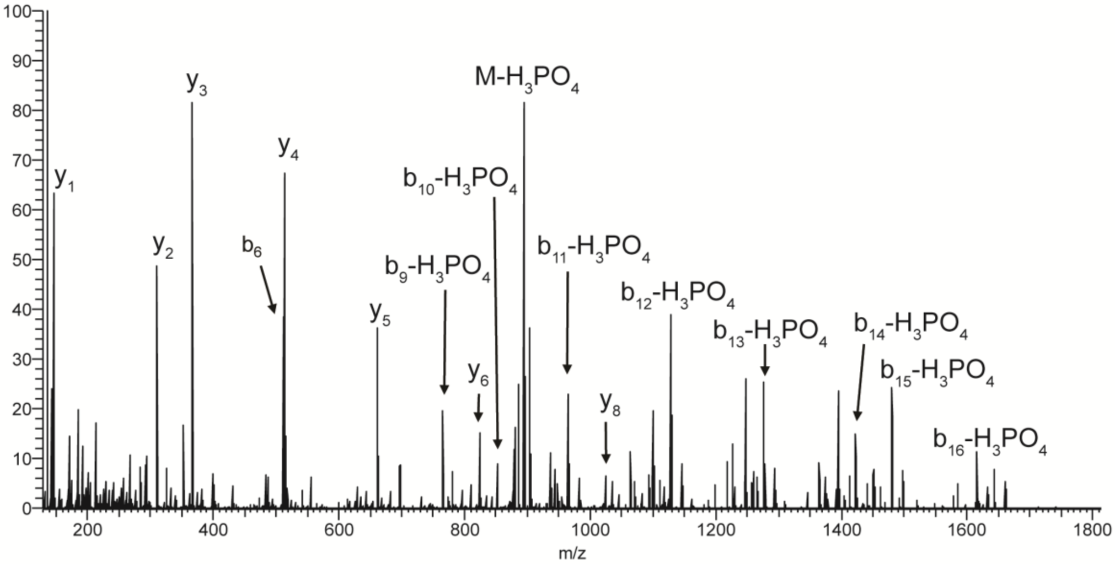

b

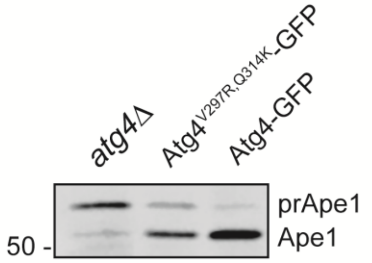

Supplementary Figure 2. Atg4 phosphorylation analysis in vivo. (a) Atg4V297R,Q314K-GFP was purified and processed for protein mass spectrometry as described in Methods. Extracted ion chromatogram of the phosphorylated GIAGGRPSSSLYFFGYK peptide is presented. Proposed structures of the fragment ions are indicated with numbered letter. The detailed data are in Supplementary Table 2. (b) Normal progression of autophagy in cells overexpressing Atg4^{V297R,Q314K}-GFP was assessed by monitoring maturation of precursor Ape1 (prApe1) into Ape1 by western blot. The *atg4Δ* strain transformed with an empty verctor (pRS416, *atg4Δ*), pTEFATG4-GFP(416) or pTEFAtg4V297R,Q314K-GFP(416), was grown to an exponential phase before precipitating proteins with trichloroacetic acid and separate them by SDS-PAGE. Western blot membranes were probed with anti-Ape1 antibodies.

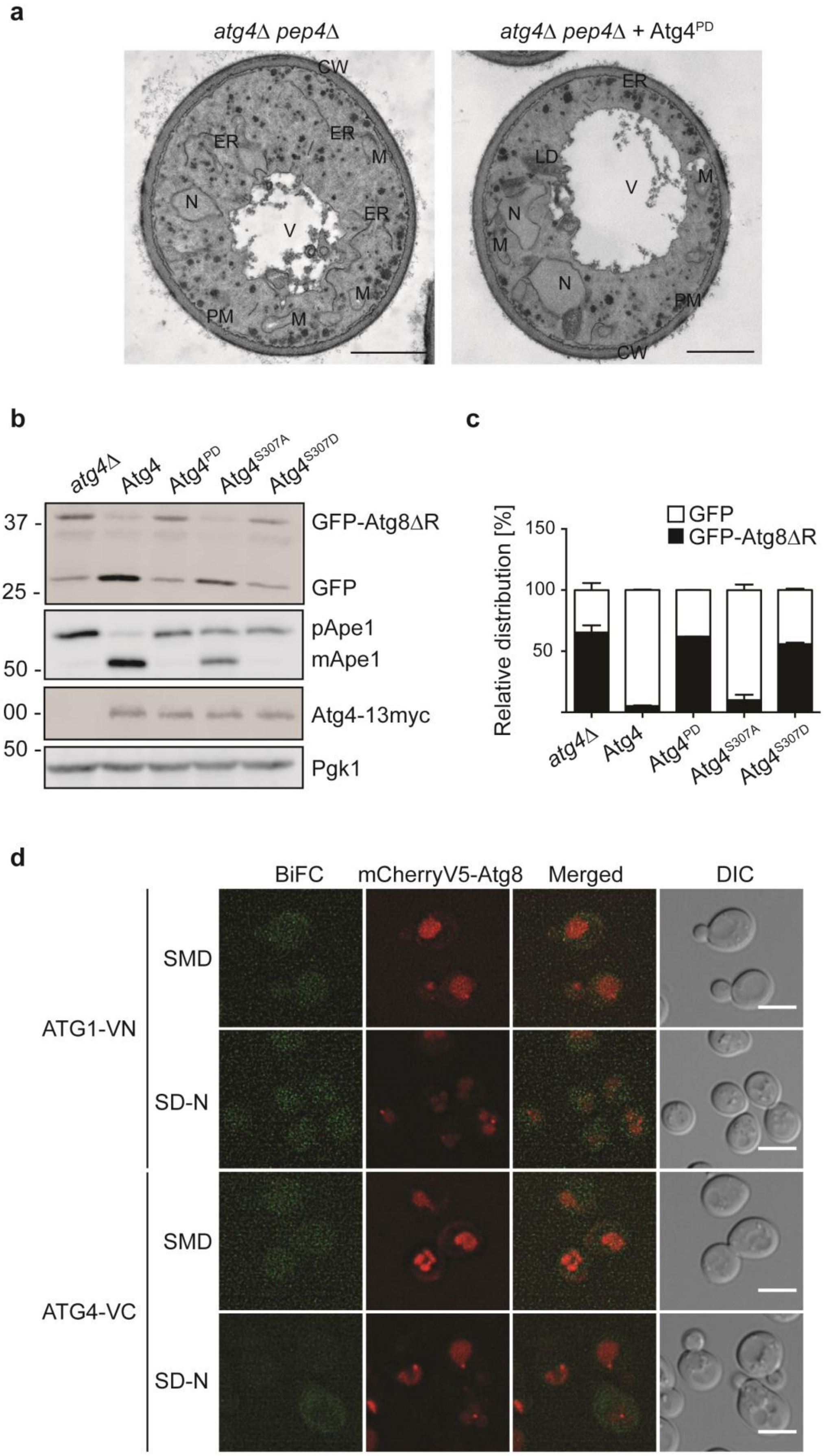
a
atg4Δ pep4Δ
atg4Δ pep4Δ + Atg4PD
CW
ER
M
N
V
PM
LD
b
atg4Δ
Atg4
Atg4PD
Atg4S307A
Atg4S307D
37
25
50
100
50
GFP-Atg8ΔR
GFP
pApe1
mApe1
Atg4-13myc
Pgk1
c
GFP
GFP-Atg8ΔR
Relative distribution [%]
150
100
50
d
BiFC
mCherryV5-Atg8
Merged
DIC
ATG1-VN
ATG4-VC
SMD
SD-N

Supplementary Figure 3. Control experiments for electron microscopy, GFP-Atg8ΔR distribution analyses and BiFC assay. (a) The *atg4Δ pep4Δ* strain transformed with an empty plasmid (JSY163) or expressing $Atg4^{PD}$-GFP (SAY145) was processed for electron microscopy as in Fig. 1d. CW, cell wall; ER, endoplasmic reticulum; LD, Lipid droplet; M, mitochondrion; N, nucleus; PM, plasma membrane; V, vacuole. Scale bar, 1 µm. (b) TCA-precipitated proteins from the cells analyzed in Fig. 2b were examined by western blot. (c) Quantification of the amounts of GFP-Atg8ΔR and GFP in the western blot shown in (b). Error bars represent the SD of 3 independent experiments. (d) Control experiments for the BiFC assay. WT cells expressing endogenous Atg1-VN (JSY181) or Atg4-VC (JSY184) and carrying the pCumCherryV5ATG8(416) plasmid were processed as in Fig. 4a. DIC, differential interference contrast. Scale bar, 5 µm.

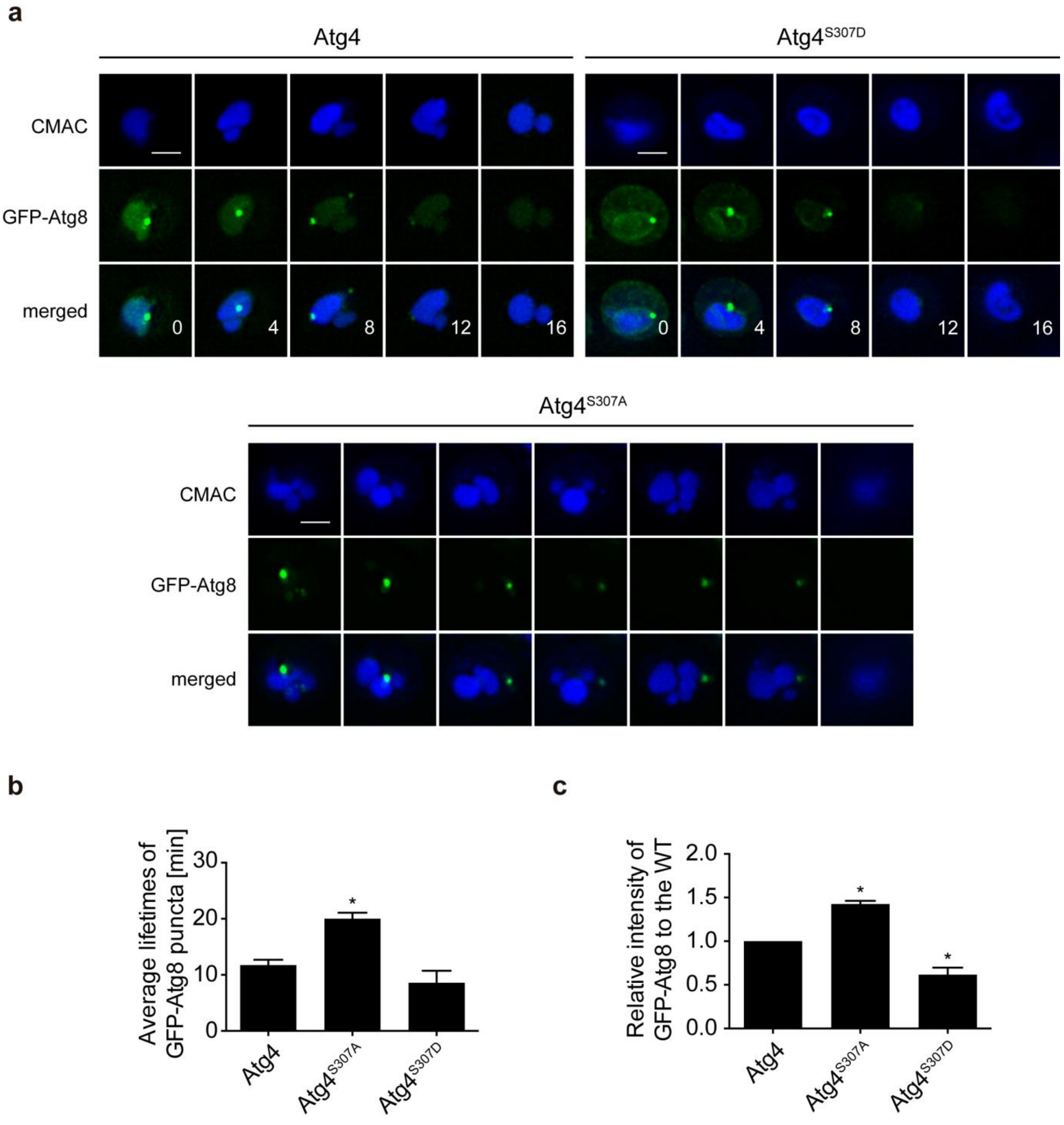

Supplementary Figure 4. Time-lapse imaging of GFP-Atg8DR-positive autophagosomes in presence of Atg4, Atg4^{S307D} and Atg4^{S307A}. (a) Cells expressing GFP-Atg8ΔR and Atg4 (RGS306), Atg4^{S307D} (RSY298) or Atg4^{S307A} (RGS297) were grown in YPD medium at 30˚C to a logarithmic phase before being after nitrogen starved in SD-N medium for 30 min. Cells were also incubated with the CMAC dye to label the vacuole, 10 min before being analysed by live-cell imaging as described in Methods with picture collected every 30 s. A representative event for each strain is shown in Supplementary Movies 1, 2 and 3. The single

focal plane frames from these videos collected at the intervals of 4 min, are shown in the panel. Scale bar: 2 µm. (b) Quantification of GFP-Atg8ΔR-positive puncta lifetimes imaged in (a). Autophagosome formation time was define and measured from the appearance until disappearance of GFP-Atg8ΔR punctum over-time. Error bars indicate the SD of three independent experiments and in each experiment, 25 cells where the PAS was monitored continuously in the same confocal planes were analyzed. Significant differences ($p<0.01$) between the various Atg4 mutants and the WT are indicated with the symbol *. (c) Fluorescence intensity quantification of GFP-Atg8ΔR punctate structures in the strains examined in (a). The average intensity of cells expressing WT Atg4 was set to 1 as relative reference. Error bars indicate the SD of three independent experiments. Significant differences ($p<0.01$) between the various Atg4 mutants and the WT are indicated with the symbol * and were calculated with the paired two-tailed Student's *t*'test.

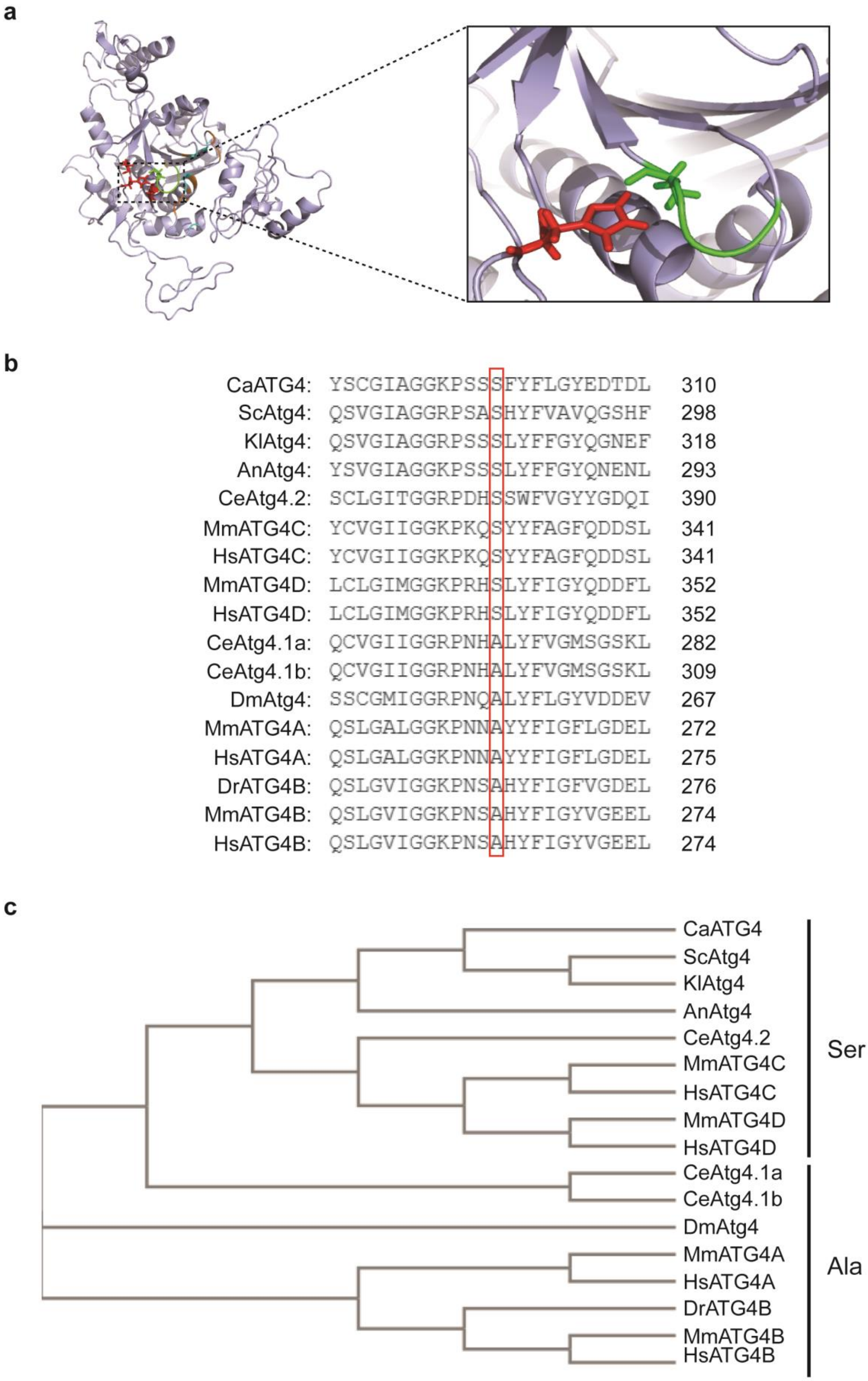
a
b
CaATG4: YSCGIAGGKPSSSFYFLGYEDTDL 310
ScAtg4: QSVGIAGGRPSASHYFVAVQGSHF 298
KlAtg4: QSVGIAGGRPSSSLYFFGYQGNEF 318
AnAtg4: YSVGIAGGKPSSSLYFFGYQNENL 293
CeAtg4.2: SCLGITGGRPDHSSWFVGYYGDQI 390
MmATG4C: YCVGIIGGKPKQSYYFAGFQDDSL 341
HsATG4C: YCVGIIGGKPKQSYYFAGFQDDSL 341
MmATG4D: LCLGIMGGKPRHSLYFIGYQDDFL 352
HsATG4D: LCLGIMGGKPRHSLYFIGYQDDFL 352
CeAtg4.1a: QCVGIIGGRPNHALYFVGMSGSKL 282
CeAtg4.1b: QCVGIIGGRPNHALYFVGMSGSKL 309
DmAtg4: SSCGMIGGRPNQALYFLGYVDDEV 267
MmATG4A: QSLGALGGKPNNAYYFIGFLGDEL 272
HsATG4A: QSLGALGGKPNNAYYFIGFLGDEL 275
DrATG4B: QSLGVIGGKPNSAHYFIGFVGDEL 276
MmATG4B: QSLGVIGGKPNSAHYFIGYVGEEL 274
HsATG4B: QSLGVIGGKPNSAHYFIGYVGEEL 274
c
CaATG4
ScAtg4
KlAtg4
AnAtg4
CeAtg4.2
MmATG4C
HsATG4C
MmATG4D
HsATG4D
CeAtg4.1a
CeAtg4.1b
DmAtg4
MmATG4A
HsATG4A
DrATG4B
MmATG4B
HsATG4B
Ser
Ala

Supplementary Figure 5. Predicted three-dimensional model of yeast Atg4 structure and conservation of S307 among eukaryotes. (a) Three-dimensional model predicting yeast Atg4 structure generated from the crystal structure of human ATG4B using the software at http://robetta.bakerlab.org/ (left) and an enlargement of the inset highlighting the region around S307 (right). The putative catalytic site (C147, D322 and H324) of Atg4 is highlighted in red. The putative regulatory loop is colored in green and conserved residues forming part of the predicted Atg8 interaction region are in light blue and orange. (b) Alignment of multiple amino acid sequences of Atg4 from different species was performed using the Clustal Omega software (http://www.ebi.ac.uk/Tools/msa/clustalo/). The residues in the equivalent position to the S307 of yeast Atg4 are highlighted by a red square. UniprotKB accession numbers are: *C. albicans* Atg4 (Q59UG3), *A. nidulans* Atg4 (Q5B7L0), *S. cerevisiae* Atg4 (P53867), *K. lactis* Atg4 (Q6CQ60), *C. elegans* Atg4.1a (Q9N30), *C. elegans* Atg4.1b (K8ESC5), *C. elegans* Atg4.2 (Q9U1N6), *D. melanogaster* Atg4 (M9PBM3), *D. rerio* Atg4B (Q6DG88), *M. musculus* ATG4A (Q8C9S8), *M. musculus* ATG4B (Q8BGE6), *M. musculus* ATG4C (Q811C2), *M. musculus* ATG4D (Q8BGV9), *H. sapiens* ATG4A (Q8WYN0), *H. sapiens* ATG4B (Q9Y4P1), *H. sapiens* ATG4C (Q96DT6) and *H. sapiens* ATG4D (Q86TL0). (c) Cladogram showing the phylogenetic relation among the Atg4 proteins from different eukaryotes obtained using the Clustal Omega software (http://www.ebi.ac.uk/Tools/msa/clustalo/). On the right of the figure it is indicated which Atg4 homologues have a serine (Ser) or an alanine (Ala) residue in the equivalent position to the S307 of yeast Atg4.

a

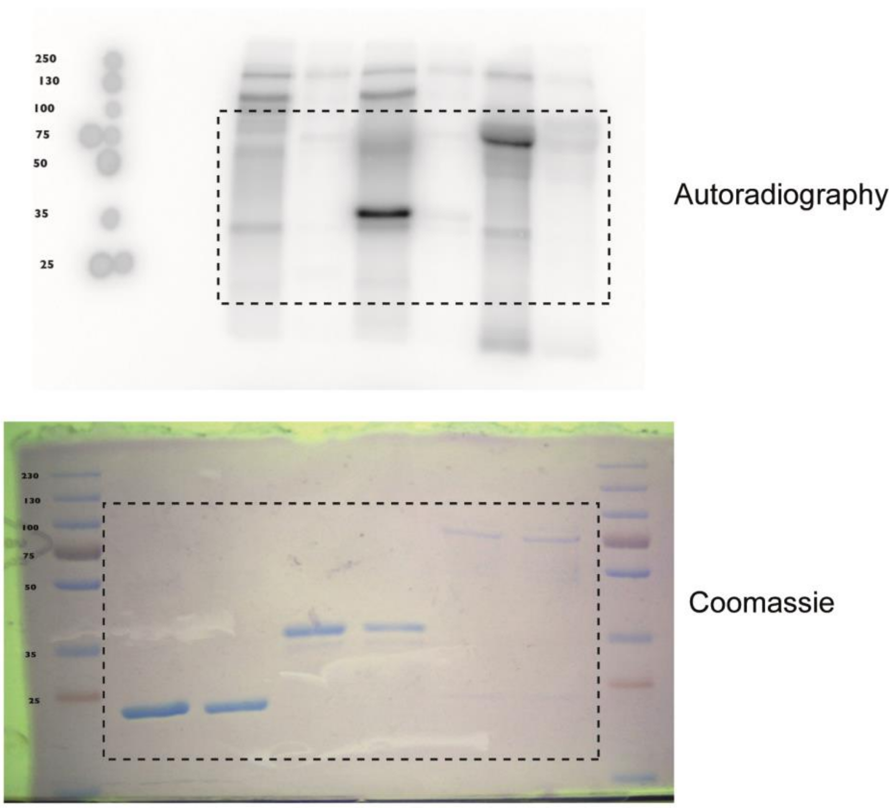
250
130
100
75
50
35
25
Autoradiography
Coomassie

b

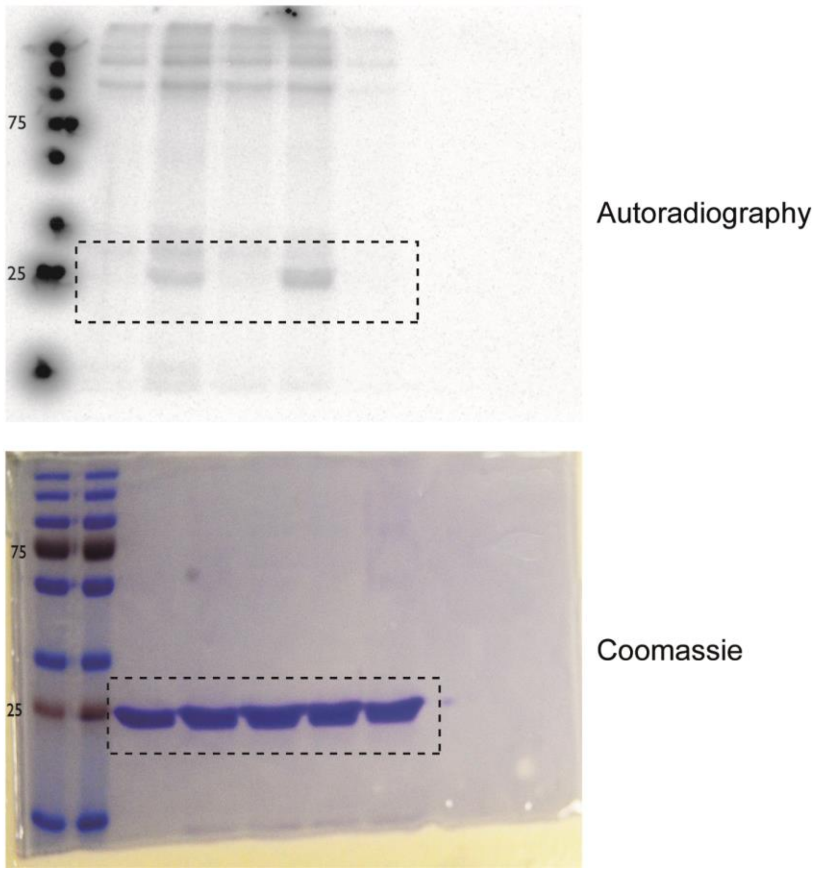
75
25
Autoradiography
75
25
Coomassie

Supplementary Figure 6. Original material. (a) Autoradiography and scan used to generate Figure 1a. (b) Autoradiography and scan used to generate Figure 1c.

a

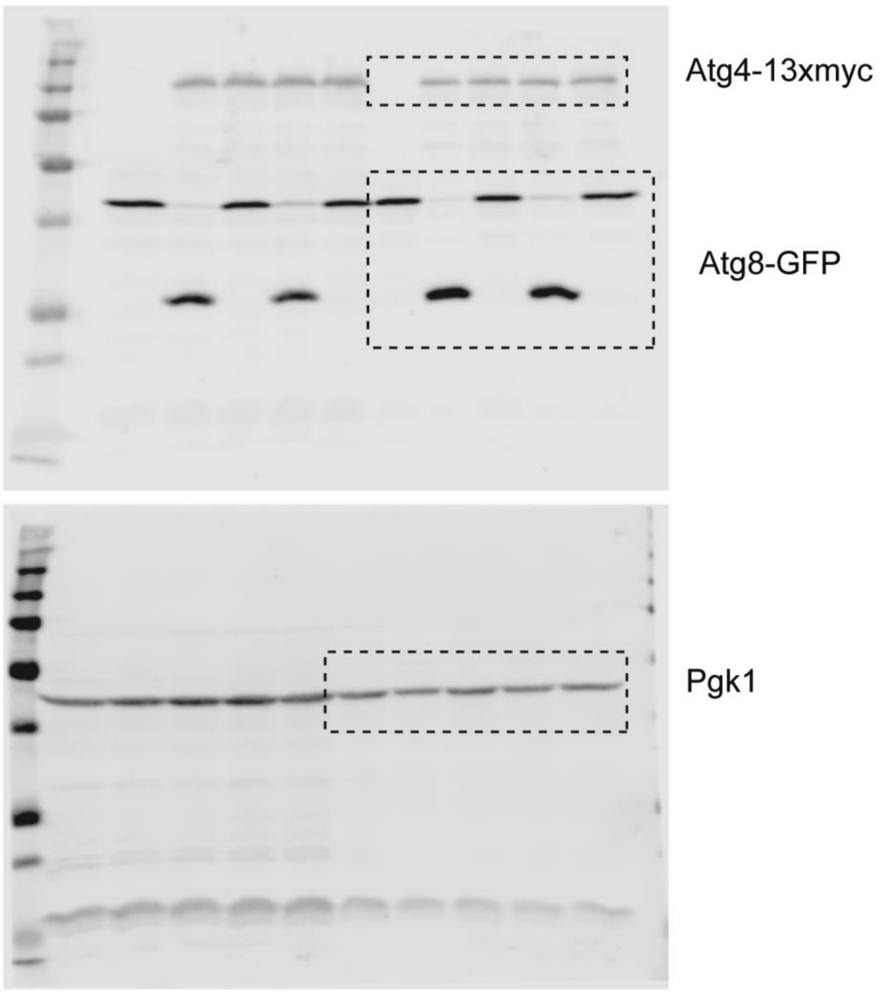

b

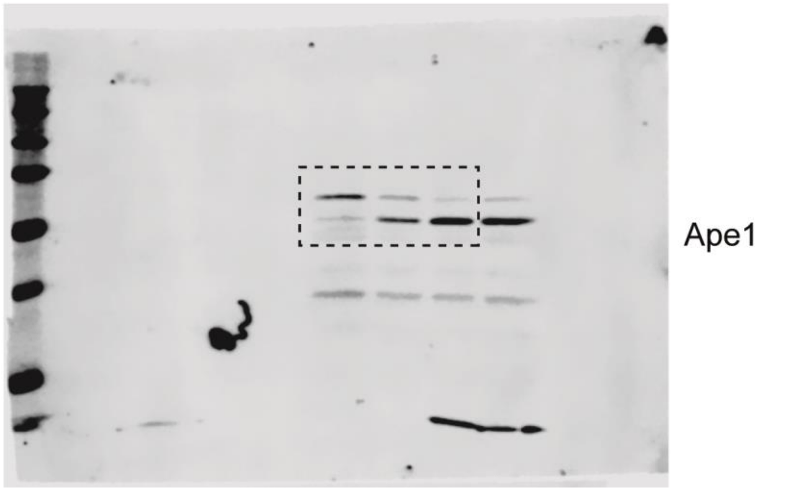

Supplementary Figure 7. Original material. (a) Scans of the western blots used to generate Figure 2a. (b) Scan of the western blots used to generate Supplementary Figure 2b.

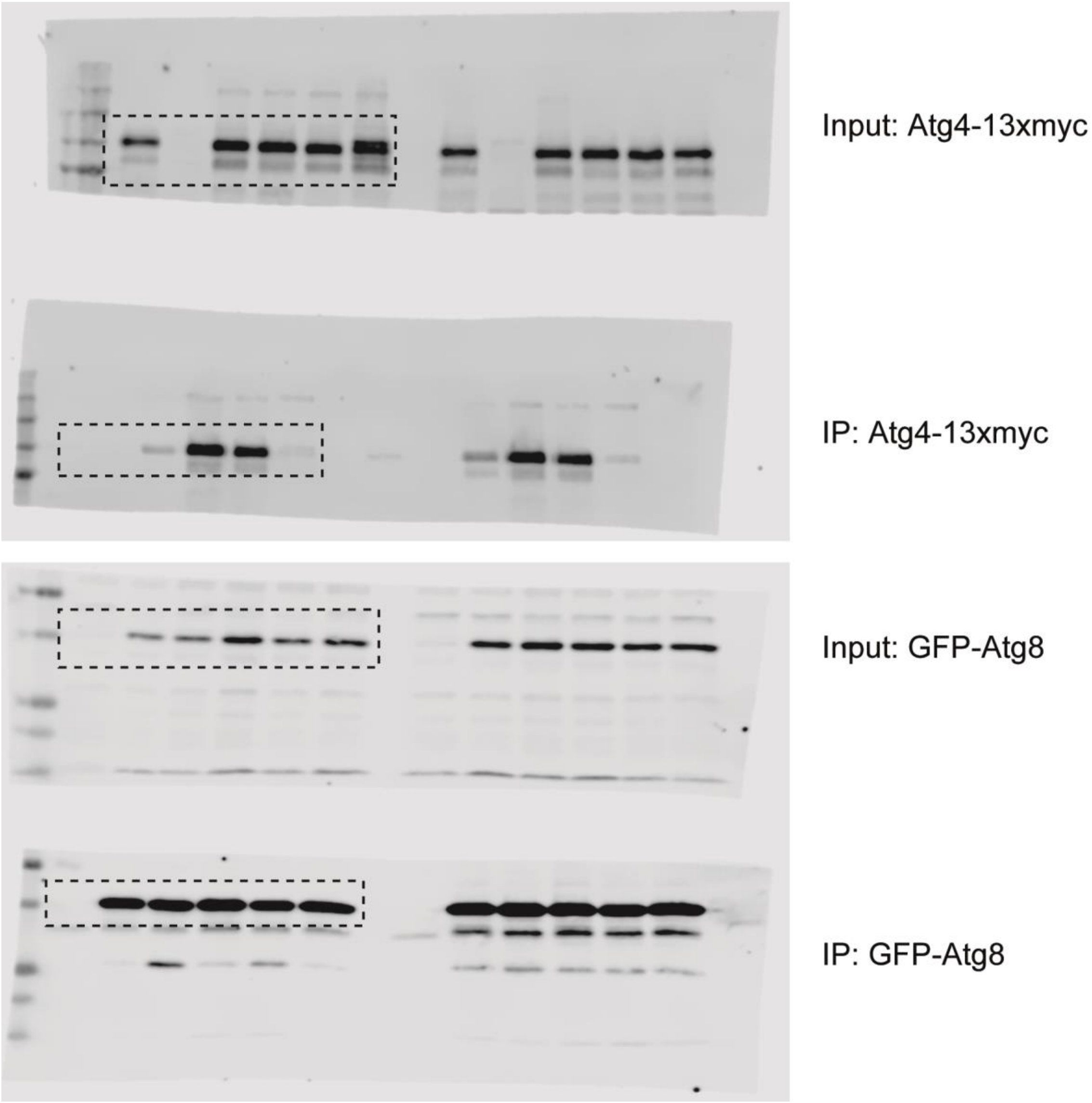

Supplementary Figure 8. Original material. Scans of the western blots used to generate Figure 3b.

Supplementary Table 1. Yeast strains used in this work

**Name**	**Genotype**	**Origin**
JSY151	SEY6210 *atg4Δ*::*TRP1 CUP1pr-GFP-ATG8ΔR*::*LEU2*	This study
JSY163	SEY6210 *atg4Δ*::*TRP1 pep4*Δ::*LEU2 pRS::URA3*	This study
JSY164	SEY6210 *atg4Δ*::*TRP1 pep4*Δ::*LEU2 ATG4*^{S307A}*-GFP*::*URA3*	This study
JSY165	SEY6210 *atg4Δ*::*TRP1 pep4*Δ::*LEU2 ATG4*^{S307D}*-GFP*::*URA3*	This study
JSY181	SEY6210 *ATG1-VN*::*HIS3*	This study
JSY184	SEY6210 *ATG4-VC*::*TRP1*	This study
JSY185	SEY6210 *ATG1-VN*::*HIS3 ATG4-VC*::*TRP1*	This study
JSY190	SEY6210 *atg2*Δ::*hphNTI ATG1-VN*::*HIS3 ATG4-VC*::*TRP1*	This study
JSY215	SEY6210 *atg2*Δ::*hphNTI atg13::LEU2 ATG1-VN*::*HIS3 ATG4-VC*::*TRP1*	This study
MNY006	SEY6210 *ATG4-GFP*::*TRP1*	This study
PJ69-4A	MATa leu2-3,112 trp1-901 ura3-52 his3-200 gal4 gal80 LYS2::GAL1-HIS3 GAL2-ADE2 met2::GAL7-lacZ	[1]
RGS297	SEY6210 *atg4Δ*::*TRP1 CUP1pr-GFP-ATG8ΔR*::*LEU2 pATG4*^{S307A}*::URA3*	This study
RGS298	SEY6210 *atg4Δ*::*TRP1 CUP1pr-GFP-ATG8ΔR*::*LEU2 pATG4*^{S307D}*::URA3*	This study
RGS306	SEY6210 *atg4Δ*::*TRP1 CUP1pr-GFP-ATG8ΔR*::*LEU2 pATG4::URA3*	This study
SAY084	SEY6210 *atg4Δ*::*TRP1*	This study
SAY114	SEY6210 *atg4Δ*::*TRP1 CUP1-ATG8-GFP*::*HIS3*	This study
SAY130	YTS159 *atg4Δ*::*HIS5 S.p*	This study
SAY144	SEY6210 *atg4Δ*::*TRP1 pep4*Δ::*LEU2 ATG4-GFP*::*URA3*	This study
SAY145	SEY6210 *atg4Δ*::*TRP1 pep4*Δ::*LEU2 ATG4*^{PD}*-GFP*::*URA3*	This study
SEY6210	*MATα ura3-52 leu2-3,112 his3-*Δ*200 trp1-*Δ*901 lys2-801 suc2-*Δ *mel GAL*	[2]
YTS159	*MATα his3*Δ*1 leu2*Δ*0 lys2*Δ*0 ura3*Δ*0 pho13*Δ::*KAN pho8*::*PHO8*Δ*60*	[3]

Supplementary Table 2. Fragments of the phosphopeptide containing S307

Ion	Theoretical mass	Charge	Fragment	Error (ppm)	Observed mass	Intensity
y1	147,1128	1	y.K.	1,1	147,112965	29455,555
b2	171,1128	1	.GI.a	1,1	171,112991	6784,47
y2	310,1761	1	g.YK.	0,4	310,176245	22795,92
y3	367,1976	1	f.GYK.	0,4	367,19776	38706,972
b6	512,294	1	.GIAGGR.p	1,3	512,294621	18049,535
y4	514,266	1	f.FGYK.	1,2	514,266646	31481,249
y5	661,3344	1	y.FFGYK.	1,1	661,335124	16951,494
b9 -H3PO4	765,4002	1	.GIAGGRPSS.s [1xPhospho]	0,8	765,400814	9385,1363
y6	824,3978	1	l.YFFGYK.	-3,5	824,394839	7189,8417
b10 -H3PO4	852,4322	1	.GIAGGRPSSS.l [1xPhospho]	2,8	852,434598	4239,2025
a11 -H3PO4	937,5214	1	.GIAGGRPSSSL.y [1xPhospho]	5	937,526105	5226,2504
b11 -H3PO4	965,5163	1	.GIAGGRPSSSL.y [1xPhospho]	1,8	965,518006	10765,566
y8	1024,5138	1	s.SLYFFGYK.	1,4	1024,515319	3141,1023
b11	1063,4932	1	.GIAGGRPSSSL.y [1xPhospho]	1,2	1063,494449	5405,7069
y9 -H2O	1093,5353	1	s.SSLYFFGYK.	1,9	1093,537427	3142,459
a12 -H3PO4	1100,5847	1	.GIAGGRPSSSLY.f [1xPhospho]	0,4	1100,58518	9392,8115
b12 -H3PO4	1128,5796	1	.GIAGGRPSSSLY.f [1xPhospho]	1	1128,580742	18525,648
b12	1226,5565	1	.GIAGGRPSSSLY.f [1xPhospho]	0,9	1226,557631	6077,6881
a13 -H3PO4	1247,6531	1	.GIAGGRPSSSLYF.f [1xPhospho]	0,7	1247,654062	12089,635
b13 -H3PO4	1275,648	1	.GIAGGRPSSSLYF.f [1xPhospho]	0,9	1275,649207	12113,115
b13	1373,6249	1	.GIAGGRPSSSLYF.f [1xPhospho]	2,5	1373,6284	2952,5813
a14 -H3PO4	1394,7215	1	.GIAGGRPSSSLYFF.g [1xPhospho]	1	1394,722916	11252,16
b14 -H3PO4	1422,7165	1	.GIAGGRPSSSLYFF.g [1xPhospho]	1,6	1422,718782	7187,6221
a15 -H3PO4	1451,743	1	.GIAGGRPSSSLYFFG.y [1xPhospho]	-0,4	1451,742493	3650,5025
b15 -H3PO4	1479,7379	1	.GIAGGRPSSSLYFFG.y [1xPhospho]	1,2	1479,739648	11264,852
b15	1577,7148	1	.GIAGGRPSSSLYFFG.y [1xPhospho]	2,3	1577,718519	1173,9552
a16 -H3PO4	1614,8063	1	.GIAGGRPSSSLYFFGY.k [1xPhospho]	0,9	1614,807866	5342,8832
b16 -H3PO4	1642,8013	1	.GIAGGRPSSSLYFFGY.k [1xPhospho]	0,4	1642,801956	3685,3678

Supplementary References

1. James, P., Halladay, J. & Craig, E.A. Genomic libraries and a host strain designed for highly efficient two-hybrid selection in yeast. *Genetics.* **144**, 1425-1436 (1996).

2. Robinson, J.S., Klionsky, D.J., Banta, L.M. & Emr, S.D. Protein sorting in *Saccharomyces cerevisiae*: isolation of mutants defective in the delivery and processing of multiple vacuolar hydrolases. *Mol Cell Biol* **8**, 4936-4948 (1988).

3. Noda, T., Matsuura, A., Wada, Y. & Ohsumi, Y. Novel system for monitoring autophagy in the yeast *Saccharomyces cerevisiae*. *Biochem Biophys Res Commun* **210**, 126-132 (1995).
